# Geographic miss and stent graft restenosis in hemodialysis access

**DOI:** 10.1080/0886022X.2026.2717813

**Published:** 2026-08-24

**Authors:** Cheng-Wei Lien, Ming-Chien Hsieh, Mu-Yang Hsieh, Meng-Kan Chen, Chih-Cheng Wu

**Affiliations:** ^a^ Cardiovascular Center, National Taiwan University Hospital, Hsin-Chu Hospital, Hsin-Chu, Taiwan; ^b^ Department of Radiology, National Taiwan University Hospital, Hsin-Chu Hospital, Hsin-Chu, Taiwan; ^c^ College of Medicine, National Taiwan University, Taipei, Taiwan; ^d^ Department of Family Medicine, National Taiwan University Hospital, Hsin-Chu Hospital, Hsin-Chu, Taiwan; ^e^ Institute of Biomedical Engineering, National Tsing-Hua University, Hsin-Chu, Taiwan; ^f^ Institute of Cellular and System Medicine, National Health Research Institutes, Zhunan, Taiwan

**Keywords:** Hemodialysis, vascular access, stent graft, restenosis, geographic miss, angioplasty

## Abstract

Stent grafts are widely used to treat dysfunctional hemodialysis vascular access, yet restenosis remains common and its predictors are incompletely understood. We analyzed 203 unique patients who underwent stent graft implantation between 2018 and 2022 from a prospectively maintained database. Geographic miss was defined as inadequate lesion coverage or suboptimal stent sizing; longitudinal geographic miss (LGM) was defined as residual stenosis >30% within 5 mm of either stent edge. The outcomes were loss of stent graft patency and access primary patency, assessed using Kaplan–Meier analysis and Cox regression models. Among 203 patients, 81% had arteriovenous grafts and 19% had arteriovenous fistulas. One-year stent graft patency and access primary patency were 63.1% and 22.7%, respectively. In the primary exploratory multivariable model, LGM was associated with both loss of stent graft patency (HR 2.62, 95% CI 1.34–5.12) and loss of access primary patency (HR 2.76, 95% CI 1.50–5.05). Other factors associated with patency outcomes included diabetes mellitus, thrombosis presentation, vessel rupture or dissection, smaller stent diameter, and stent graft length. LGM remained associated with both outcomes in an additional clinically adjusted model. These findings suggest that LGM may represent a potentially modifiable procedural target, although prospective studies with standardized imaging follow-up and prospective validation are needed.

## Graphical Abstract

**Figure:**
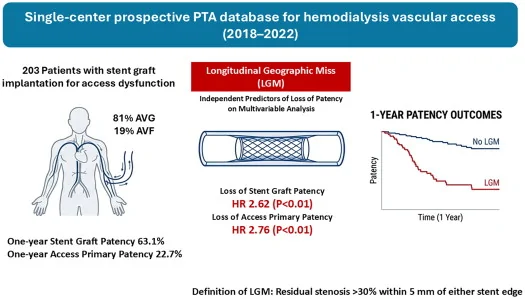

## Introduction

A well-functioning vascular access is essential for hemodialysis, yet dysfunction due to stenosis and thrombosis remains a major cause of morbidity and access loss in these patients [1]. Percutaneous transluminal angioplasty (PTA) has long been the standard treatment, but its durability is limited, with frequent restenosis requiring repeated interventions [2]. To overcome these limitations, stent grafts have emerged as an important device that improves post-PTA patency in hemodialysis vascular access. Randomized controlled trials have demonstrated that stent grafts significantly prolong primary patency compared with angioplasty alone in arteriovenous graft (AVG) anastomotic stenosis, cephalic arch stenosis, and in-stent restenosis [3–6]. Despite these advantages, stent graft restenosis remains a significant challenge, particularly at the stent edges. In pivotal trials, 40–50% of stent grafts developed restenosis or required re-intervention within 12 months, with most failures attributable to edge-related neointimal hyperplasia [4]. Edge restenosis is clinically important because it may reflect the interaction between residual lesion, landing-zone selection, vessel injury, and neointimal hyperplasia after stent graft deployment.

Longitudinal geographic miss is a measurable procedural factor that may be especially relevant to edge restenosis. It describes residual stenosis adjacent to the stent edge, suggesting incomplete coverage of the diseased segment. Although geographic miss has been studied in coronary interventions [7], its relevance to hemodialysis access circuits has not been systematically evaluated. This knowledge gap is important because dialysis access differs from coronary arteries in flow conditions, lesion distribution, repeated puncture, venous remodeling, and thrombosis risk. Moreover, longitudinal geographic miss may be potentially modifiable through improved lesion assessment and landing-zone selection, but it may also reflect lesion complexity or operator decision-making.

Therefore, this study aimed to identify clinical and angiographic predictors of stent graft restenosis and overall vascular access patency in a real-world hemodialysis cohort, with particular attention to longitudinal geographic miss as a measurable and potentially modifiable technical factor.

## Materials and methods

### Data collection

This was a retrospective analysis of stent graft implantation in hemodialysis vascular access performed between July 1, 2018, and December 31, 2022. Data were obtained from a prospectively maintained institutional percutaneous transluminal angioplasty (PTA) database at National Taiwan University Hospital Hsinchu Branch. This database is not publicly available and is maintained for clinical and research purposes within the hospital. Cases were identified by filtering for procedures coded with ‘stent graft’. Clinical data (age, sex, comorbidities, dialysis duration), access characteristics (shunt age, type, and location), lesion features (site, severity, and length), and procedural details were systematically extracted from standardized angioplasty reports. For patients who underwent more than one stent graft procedure during the study period, only the first eligible stent graft procedure was included as the index procedure. Subsequent reinterventions or repeat stent graft procedures after the index procedure were recorded as follow-up outcomes but were not entered as additional index observations. Thus, the final analytic cohort included one index stent graft procedure per patient. This study was conducted in accordance with the principles of the Declaration of Helsinki. The study protocol was approved by the Institutional Review Board of National Taiwan University Hospital Hsinchu Branch (approval number: 111-120-E). The requirement for informed consent was waived due to the retrospective nature of the study and the use of de-identified data.

### Angiography analysis

Stored digital angiograms were reviewed using a dedicated workstation (Digital DLX; GE Healthcare, Buc, France). Reference vessel diameter was defined as the adjacent normal vein upstream of the lesion. Stenosis severity was expressed as the percentage reduction of minimal lumen diameter relative to reference. ‘Geographic miss’ (GM) was defined according to prior coronary literature [7]. Longitudinal GM (LGM) referred to residual stenosis >30% within 5 mm of either stent edge, an operational threshold adapted from coronary restenosis studies because no validated dialysis-access-specific definition is available [8,9]. The threshold was used to classify the immediate post-deployment landing-zone condition and was not intended as a criterion for clinical reintervention. Axial GM was defined as stent/vessel size ratio <0.9 (under-sizing) or >1.1 (over-sizing). Angiographic evaluation was initially performed by an experienced radiographic technician who was blinded to clinical outcomes. To assess measurement reproducibility, a sample of 30 angiograms was reevaluated. For intraobserver agreement, the original radiographic technician repeated the assessment while blinded to clinical outcomes and the original classification. For interobserver agreement, a second physician investigator independently reviewed the same angiograms while blinded to clinical outcomes and the original assessment. Agreement for LGM classification was assessed using Cohen’s κ. Borderline cases were adjudicated by joint review, and the final classification was determined by consensus according to the prespecified LGM definition.

### Endovascular procedure

All procedures were performed by experienced interventional nephrologists or radiologists using standard endovascular techniques [10]. The target lesion was first traversed with a 0.035-inch hydrophilic guidewire (Terumo, Tokyo, Japan), followed by initial balloon angioplasty. The balloon catheter was selected according to the reference vessel diameter or up to 1 mm larger. Initial balloon inflation was performed at nominal pressure; if a residual balloon waist persisted, the inflation pressure was increased stepwise until waist resolution or until the rated burst pressure was reached. Stent graft placement was considered after angioplasty according to clinical indication, angiographic findings, reimbursement criteria, and operator judgment. During the study period, the available stent grafts included the Viabahn Endoprosthesis (W. L. Gore, Flagstaff, AZ, USA) and the Covera Vascular Covered Stent (Bard Peripheral Vascular, Tempe, AZ, USA). Device selection was determined by operator preference. In Taiwan, stent grafts were reimbursed for vessel rupture or recurrent graft–venous junction restenosis within 3 months after intervention. Stent graft diameter was selected based on the maximal diameter of the access vessel adjacent to the lesion. Deployment across frequent puncture zones was avoided whenever possible to reduce the risk of stent migration or fracture. At the end of the procedure, technical success was confirmed by restoration of brisk antegrade flow on fistulography and the presence of a palpable continuous thrill.

### Follow-up protocol

Patients were followed at their dialysis centers under a standardized surveillance program, including physical examination, access flow assessment, and venous pressure monitoring. Suspicion of dysfunction prompted referral for repeat imaging and intervention. Additional procedures were performed if >50% stenosis was detected with concordant clinical or hemodynamic abnormalities, in accordance with NKF-KDOQI guidelines [11]. Follow-up information was collected by a dedicated coordinator through telephone contact at 3-month intervals for one year, and all procedure notes and angiograms were reviewed retrospectively to verify outcomes.

### Outcomes

All patency outcomes were calculated from the date of the index stent graft procedure. Repeat endovascular procedures, including repeat angioplasty or repeat stent graft placement after the index procedure, were treated as follow-up events according to the relevant patency definitions and were not counted as additional index procedures.

The primary outcome was stent graft patency, defined as the time from the index intervention to clinically detected and angiographically confirmed restenosis >50% at either edge of the stent graft. Access primary patency was defined as the time from index intervention to access thrombosis or any repeat intervention. Access assisted primary patency was defined as the time from index intervention to access thrombosis or surgical revision, with allowance for repeat endovascular procedures. Secondary patency was defined as the time from index intervention to permanent access abandonment or surgical revision. For time-to-event analyses, patients without the event of interest were censored at death, kidney transplantation, loss to follow-up, or completion of the one-year follow-up period, whichever occurred first. Access abandonment and surgical revision were treated as patency-loss events for access primary patency if they occurred before another qualifying event. For assisted primary patency and secondary patency, surgical revision and permanent access abandonment were handled according to the corresponding endpoint definitions [12].

### Statistical analysis

Continuous variables were summarized as mean ± standard deviation or median with interquartile range, as appropriate. Categorical variables were presented as counts and percentages. Between-group comparisons were performed using Student’s t test, analysis of variance, Mann–Whitney U test, chi-square test, or Fisher’s exact test, as appropriate.

Kaplan–Meier analysis was used to estimate stent graft patency and access primary patency, and groups were compared using the log-rank test. Patients who did not experience the event of interest were censored at death, kidney transplantation, loss to follow-up, or the end of the one-year follow-up period, whichever occurred first. Cox proportional hazards regression was used to evaluate factors associated with loss of stent graft patency and loss of access primary patency. Hazard ratios greater than 1.00 indicate a higher risk of patency loss. No formal *a priori* sample size calculation was performed because this was a retrospective analysis of all eligible patients during the study period. To provide statistical context for the primary endpoint, an event-based power assessment was performed using Schoenfeld’s approximation. The study may nevertheless have been underpowered to detect smaller effect sizes and for subgroup analyses.

Because the primary aim of this retrospective exploratory study was to identify clinical, anatomical, and procedural factors associated with patency outcomes after stent graft implantation, univariable Cox regression analyses were first performed for candidate predictors. Variables with a univariable p value <0.10 were entered into the primary exploratory multivariable model for each outcome. Because LGM was the main technical factor identified in the exploratory analysis, and because it may be associated with lesion and procedural complexity, an additional LGM-focused clinically adjusted model was constructed. This model included LGM and anatomical or procedural variables that could plausibly confound the association between LGM and patency outcomes, including lesion length, stenosis severity, thrombosis presentation, rupture or dissection, stent diameter, stent length, and lesion location. Actual puncture sites and operator intent to avoid cannulation areas were not recorded. Therefore, PTFE–vein junction involvement was used as an exploratory lesion-location proxy potentially related to cannulation-zone proximity. As a sensitivity analysis, the multivariable Cox models were repeated after excluding non-Viabahn stent graft procedures. To explore potential learning-curve effects, operator experience was defined as the cumulative number of prior stent graft procedures performed by the same operator before each index procedure. Operator experience was analyzed as a continuous variable per 10 prior procedures in a sensitivity Cox model.

All analyses were performed at the patient level, with one index stent graft procedure included per patient. Subsequent reinterventions after the index procedure were treated as follow-up events and were not entered as additional index observations. Patients without available follow-up information or angiographic images were excluded before analysis; therefore, all variables required for the multivariable Cox models were complete in the final analytic cohort, and no imputation was performed. Collinearity among LGM, stent length, stent diameter, oversizing, and undersizing was assessed using variance inflation factors. Statistical significance was defined as a two-sided p value <0.05. Analyses were conducted using SPSS version 22.0 (SPSS Inc., Chicago, IL, USA).

## Results

### Baseline characteristics of study participants

A total of 224 patients who underwent stent graft implantation for hemodialysis vascular access were identified from the database. Twenty-one patients were excluded: 6 due to unavailable follow-up information, 9 due to unavailable angiographic images, 3 for central vein lesions, and 3 for aneurysms. Consequently, 203 unique patients with 203 index stent graft procedures were included in the final patient-level analysis. The mean age of the study participants was 70 years (SD, 13), and 38% were male. The median dialysis duration was 32 months (IQR 12–60), and the median shunt age was 24 months (IQR 11–43). Hypertension and diabetes mellitus were present in 65% and 55% of patients, respectively, while 44% had coronary artery disease. Most accesses were AVGs (81%), with 19% arteriovenous fistulas (AVFs). Thrombosis was the presenting indication in 49% of procedures. Stent graft implantation most commonly involved the graft–vein junction (76% of AVG cases), whereas in AVF the basilic vein was the most frequent site. The Viabahn stent was used in 92% of procedures. Slightly more than half of stents were ≤7 mm in diameter, and one-fifth were ≥15 cm in length. Longitudinal geographic miss was observed in 16% of procedures, over-sizing in 17%, and under-sizing in 9%. Other baseline details are summarized in Table 1. In the reproducibility analysis, intraobserver agreement for LGM classification was excellent, with a Cohen’s κ of 0.93. Interobserver agreement between the radiographic technician and the physician investigator was also high, with a Cohen’s κ of 0.86.

**Table 1.** Baseline characteristics of study participants.

Baseline factors	ALL		AVF		AVG	
**Number**	203	100.0%	39	19.2%	164	80.7%
**Patient factors**						
Age, y	70	13	68	13	70	12
Male, %	78	38.4%	18	46.2%	60	36.6%
HD duration, mo	32	(12, 60)	54	(28, 96)	29	(12, 48)
Hypertension	132	65.0%	27	69.2%	105	64.0%
Diabetes mellitus	112	55.2%	22	56.4%	90	54.9%
CAD	89	43.8%	19	48.7%	70	42.7%
Hyperlipidemia	51	25.1%	10	25.6%	41	25.0%
Smoking	18	8.9%	4	10.3%	14	8.5%
**Access and lesion factors**						
Shunt age, mo	24	(11,43)	35	(24, 72)	24	(9, 36)
Right-arm access	34	16.7%	10	25.6%	24	14.6%
Upper-arm access	59	29.1%	13	33.3%	46	28.0%
Thrombosis	100	49.3%	21	53.8%	79	48.2%
Lesion length ≥4 cm	110	54.2%	22	56.4%	88	53.7%
Lesion stenosis ≥90%	121	59.6%	20	51.3%	101	61.6%
Multiple lesions	46	22.7%	9	23.1%	37	22.6%
Rupture or dissection	73	36.0%	27	69.2%	46	28.0%
Stent location						
PTFE-vein junction	125	61.6%	0	0.0%	125	76.2%
Native veins	67	33.0%	39	100.0%	28	17.1%
Others	11	5.4%	0	0.0%	11	6.7%
**Procedural factors**						
Stent type						
Viabahn stent	186	91.6%	38	97.4%	148	90.2%
Covera stent	17	8.4%	1	2.6%	16	9.8%
Stent size						
Diameter ≤7 mm	112	55.2%	22	56.4%	90	54.9%
Length ≥15 cm	40	19.7%	10	25.6%	30	18.3%
Stent landing condition						
Longitudinal GM	32	15.8%	7	17.9%	25	15.2%
Over-sizing (>1.1)	34	16.7%	6	15.4%	28	17.1%
Under-sizing (<0.9)	19	9.4%	4	10.3%	15	9.1%
Operator’s prior stent procedure	24	(8, 53)	20	(4, 41)	25	(9, 54)

Values are expressed as mean (SD), median (IQR), or n (%) as appropriate.

Abbreviations: AVF, arteriovenous fistula; AVG, arteriovenous graft; CAD, coronary artery disease; HD, hemodialysis; GM, geographic miss.

### Follow-up of patency outcomes

**Figure 1.**
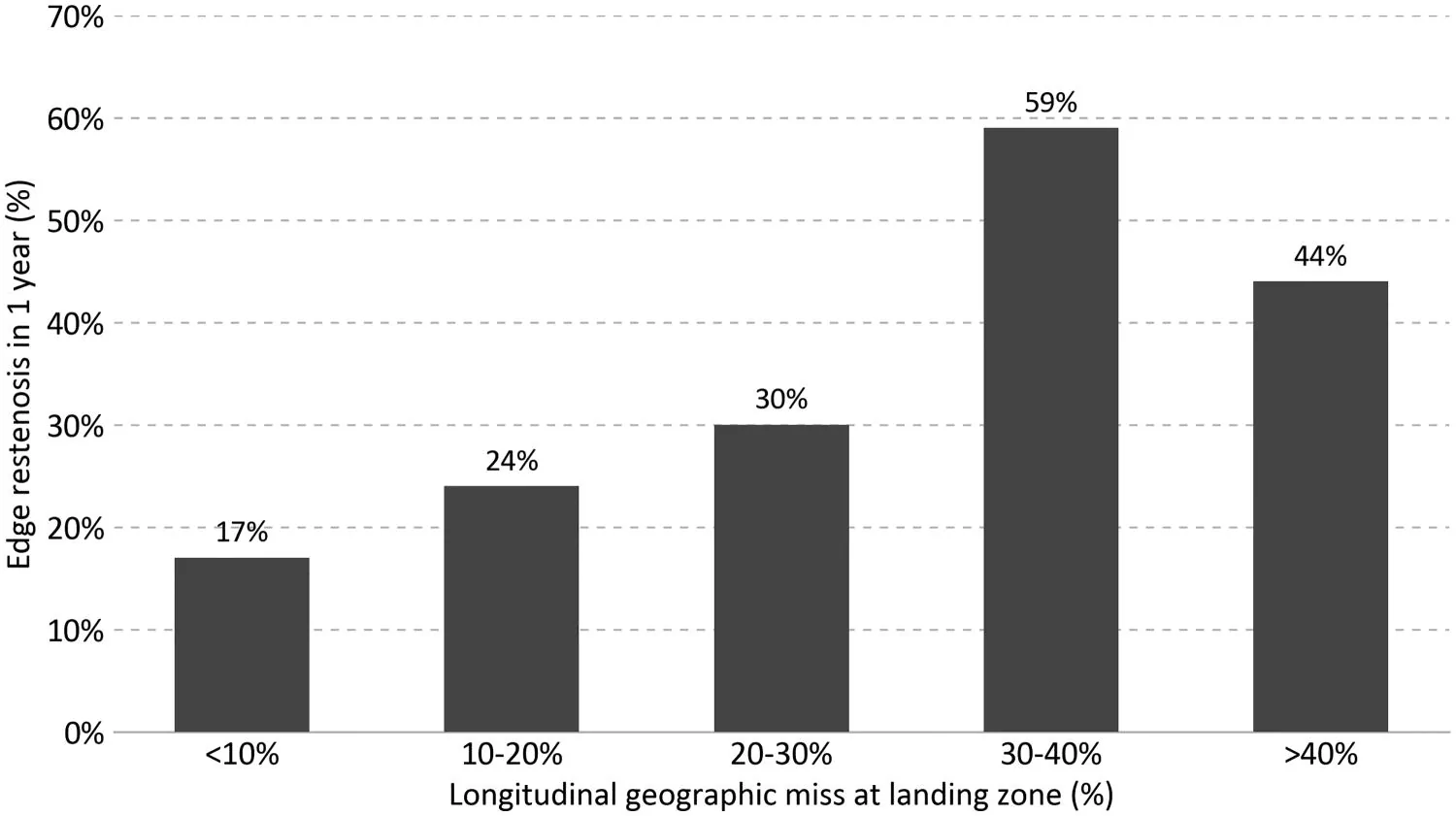
Incidence of stent edge restenosis at one year according to the severity of residual edge stenosis.

**Figure 2.**
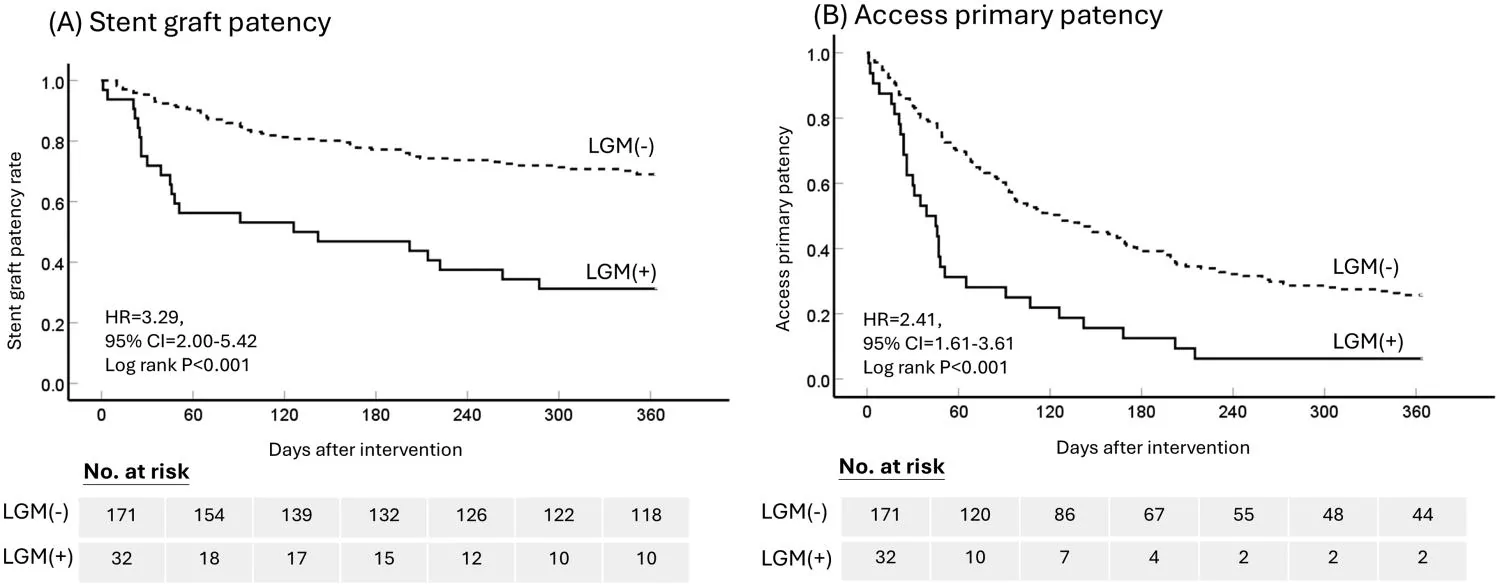
Kaplan–Meier curves of stent graft patency and access primary patency according to longitudinal geographic miss (LGM).

The median follow-up duration was 12 months (interquartile range, 3.6–12 months). During one year of follow-up, reintervention was required in 157 accesses. Eighty-five assisted-primary-patency events occurred, resulting in an assisted primary patency rate of 58.1% at one year. Access primary patency was 22.7%, while assisted primary patency and secondary patency were 58.1% and 95.1%, respectively (Table 2). Subgroup analysis showed higher primary and assisted primary patency in AVF compared with AVG. In the edge-level analysis of 406 stent graft margins, restenosis increased substantially when residual edge stenosis reached 30–40%, with a restenosis rate of 59% compared with 30% among edges with 20–30% residual stenosis (Figure 1). This finding was consistent with the clinical relevance of the prespecified >30% stenosis threshold in this cohort. Stents with LGM showed significantly lower graft and access patency compared with those without LGM (Figure 2). For the primary endpoint, 75 stent graft patency-loss events occurred during one-year follow-up, and LGM was present in 32 patients (15.8%). Based on these observed data, the event-based power assessment indicated approximately 86% power to detect the observed LGM effect size for loss of stent graft patency.

**Table 2.** Patency outcomes 1 year after intervention.

1 year patency rate	ALL (*n* = 203)		AVF (*n* = 39)		AVG (*n* = 164)	
	No. of events	Patency rate	No. of events	Patency rate	No. of events	Patency rate
Stent graft patency rate	75	63.1%	13	66.7%	62	62.2%
Access primary patency rate	157	22.7%	25	35.9%	132	19.5%
Access assisted primary patency rate	85	58.1%	7	82.1%	78	52.4%
Secondary patency rate	10	95.1%	1	97.4%	9	94.5%

Abbreviations: AVF, arteriovenous fistula; AVG, arteriovenous graft.

### Regression analysis of factors associated with patency outcomes

In univariable Cox regression, factors associated with loss of stent graft patency included diabetes mellitus, thrombosis presentation, vessel rupture or dissection, stent diameter, LGM, oversizing, and undersizing (Table 3). Factors meeting the prespecified *p* < 0.10 criterion for loss of access primary patency included diabetes mellitus, AVG access, multiple stenoses, smaller stent diameter, stent length ≥15 cm, oversizing, and LGM.

**Table 3.** Univariable cox regression analysis for loss of stent graft patency and loss of access primary patency.

	Unit of increase or	Loss of stent graft patency		Loss of access primary patency	
Factors	Reference group	HR (95% CI)	P value	HR (95% CI)	P value
**Patient factors**					
Age	1 yr	1.00 (0.98–1.02)	0.87	1.00 (0.99–1.01)	0.93
Men	Women	1.19 (0.76–1.89)	0.45	0.87 (0.63–1.20)	0.40
Hypertension	No	1.50 (0.91–2.48)	0.12	1.15 (0.83–1.61)	0.41
Diabetes mellitus	No	1.59 (0.99–2.55)	**0.05**	1.48 (1.07–2.04)	**0.02**
CAD	No	1.08 (0.68–1.70)	0.75	0.94 (0.68–1.29)	0.69
Hyperlipidemia	No	0.71 (0.40–1.25)	0.24	1.10 (0.76–1.57)	0.62
Smoking	No	0.67 (0.27–1.66)	0.38	0.73 (0.41–1.32)	0.29
**Access factors**					
Shunt age	1 yr	1.00 (1.00–1.01)	0.64	1.00 (1.00–1.00)	0.72
AVG	AVF	1.15 (0.63–2.09)	0.65	1.57 (1.03–2.41)	**0.04**
Upper-arm access	Forearm access	1.13 (0.69–1.84)	0.63	1.05 (0.74–1.48)	0.79
Lesion length ≥4 cm	<4 cm	1.24 (0.78–1.97)	0.36	0.98 (0.72–1.34)	0.90
Lesion stenosis ≥90%	<90%	1.51 (0.95–2.35)	0.11	1.20 (0.87–1.64)	0.27
Multiple stenoses	Single stenosis	1.13 (0.67–1.92)	0.65	1.44 (1.00–2.07)	**0.05**
Thrombosis presentation	Stenosis presentation	1.52 (0.96–2.41)	**0.08**	0.86 (0.63–1.17)	0.33
Vessel rupture or dissection	No	1.48 (0.94–2.33)	**0.09**	0.90 (0.65–1.26)	0.55
PTFE-vein junction	No	1.12 (0.70–1.80)	0.64	1.08 (0.78–1.49)	0.64
**Procedural factors**					
Viabahn stent	Covera stent	1.41 (0.57–3.49)	0.46	1.22 (0.69–2.16)	0.49
Stent ≤7 mm	>7 mm	1.93 (1.19–3.13)	**0.01**	0.76 (0.56–1.05)	**0.09**
Length ≥15 cm	<15 cm	1.39 (0.82–2.37)	0.22	0.67 (0.44–1.03)	**0.07**
Longitudinal GM	No	3.29 (2.00–5.42)	**<0.001**	2.41 (1.61–3.61)	**<0.001**
Over-sizing (>1.1)	No	2.34 (1.40–3.90)	**<0.01**	1.85 (1.25–2.75)	**<0.01**
Under-sizing (<0.9)	No	2.37 (1.28–4.39)	**<0.01**	1.43 (0.88–2.40)	0.15
Operator’s prior stent procedures	10 procedures	0.99 (0.94–1.05)	0.77	0.99 (0.92–1.08)	0.78

Abbreviations: AVF, arteriovenous fistula; AVG, arteriovenous graft; CAD, coronary artery disease; CI, confidence interval; GM, geographic miss; HR, hazard ratio; PTFE, polytetrafluoroethylene.

Operator’s prior stent procedures: per 10 prior stent procedures before the index procedure.

In the primary exploratory multivariable model, diabetes mellitus, thrombosis presentation, vessel rupture or dissection, stent diameter, and LGM were independently associated with loss of stent graft patency (Table 4). Specifically, diabetes mellitus was associated with a higher hazard of stent graft patency loss (HR 1.82, 95% CI 1.13–2.94, *p* = 0.01), as were thrombosis presentation (HR 1.84, 95% CI 1.11–3.05, *p* = 0.02), vessel rupture or dissection (HR 1.69, 95% CI 1.05–2.73, *p* = 0.03), stent diameter ≤7 mm (HR 1.79, 95% CI 1.08–2.96, *p* = 0.02), and LGM (HR 2.62, 95% CI 1.34–5.12, *p* < 0.01). Oversizing and undersizing were not independently associated with loss of stent graft patency after multivariable adjustment. For loss of access primary patency, LGM remained independently associated with a higher hazard of patency loss (HR 2.76, 95% CI 1.50–5.05, *p* < 0.01). In contrast, stent graft length ≥15 cm was associated with a lower hazard of access primary patency loss (HR 0.53, 95% CI 0.33–0.84, *p* < 0.01). Diabetes mellitus and AVG access showed borderline associations but did not reach statistical significance in the multivariable model. Collinearity assessment showed no evidence of substantial collinearity among LGM, stent graft length, stent graft diameter, oversizing, and undersizing, with all variance inflation factors <2.

**Table 4.** Multivariable Cox regression models for loss of stent graft patency and loss of access primary patency.

		Loss of stent graft patency		Loss of access primary patency	
Factors	Reference group	HR (95% CI)	P value	HR (95% CI)	P value
**Primary exploratory model (including variables with *p* < 0.1 in univariable analysis)**					
Diabetes mellitus	No	1.82 (1.13–2.94)	0.01	1.39 (1.00– 1.94)	0.05
AVG	AVF	NA	NA	1.50 (0.98–2.30)	0.07
Thrombosis presentation	Stenosis presentation	1.84 (1.11–3.05)	0.02	NA	NA
Rupture or dissection	No	1.69 (1.05–2.73)	0.03	NA	NA
Multiple stenoses	Single stenosis	NA	NA	1.06 (0.68–1.65)	0.81
Stent diameter ≤7 mm	Stent diameter >7 mm	1.79 (1.08–2.96)	0.02	1.29 (0.91–1.84)	0.16
Stent length ≥15 cm	Stent length <15 cm	NA	NA	0.53 (0.33– 0.84)	<0.01
Longitudinal GM	No	2.62 (1.34–5.12)	<0.01	2.76 (1.50–5.05)	<0.01
Oversize >1.1	No	1.30 (0.62–2.70)	0.49	1.14 (0.66–1.97)	0.64
Undersize <0.9	No	1.55 (0.77–3.08)	0.22	NA	NA
**Additional longitudinal GM-focused model**					
Lesion length ≥4 cm	Lesion length ≤4 cm	1.06 (0.64–1.77)	0.82	1.11 (0.80–1.56)	0.53
Stenosis ≥90%	Stenosis <90%	0.73 (0.31–1.69)	0.46	1.22 (0.68–2.20)	0.51
Thrombosis presentation	Stenosis presentation	1.33 (0.57–3.10)	0.50	0.97 (0.53–1.75)	0.91
Rupture or dissection	No	1.44 (0.79–2.64)	0.24	0.91 (0.58–1.41)	0.66
Stent diameter ≤7 mm	Stent diameter >7 mm	1.69 (1.04–2.75)	0.04	1.44 (0.99–2.09)	0.05
Stent length ≥15 cm	Stent length <15 cm	1.01 (0.55–1.84)	0.98	0.45 (0.28–0.73)	<0.01
PTFE-vein junction	No	0.98 (0.54–1.77)	0.94	0.97 (0.64–1.47)	0.87
Longitudinal GM	No	3.38 (1.96–5.83)	<0.001	3.02 (1.92– 4.73)	<0.001

Abbreviations: AVF, arteriovenous fistula; AVG, arteriovenous graft; CI, confidence interval; GM, geographic miss; HR, hazard ratio; NA, not analyzed; PTFE, polytetrafluoroethylene.

In the additional LGM-focused clinically adjusted model, LGM remained strongly associated with both loss of stent graft patency (HR 3.38, 95% CI 1.96–5.83, *p* < 0.001) and loss of access primary patency (HR 3.02, 95% CI 1.92–4.73, *p* < 0.001) after adjustment for lesion length, stenosis severity, thrombosis presentation, rupture or dissection, stent diameter, stent length, and lesion location represented by PTFE–vein junction involvement (Table 4). PTFE–vein junction involvement was included as an available proxy for lesion location potentially adjacent to common cannulation or puncture segments, because actual puncture sites and operator intent to avoid puncture zones were not recorded in the retrospective database. Stent graft length ≥15 cm also remained protective for access primary patency loss in this model (HR 0.45, 95% CI 0.28–0.73, *p* < 0.01).

In the sensitivity analysis restricted to Viabahn stent graft procedures, LGM remained significantly associated with both loss of stent graft patency (HR 2.74, 95% CI 1.38–5.43, *p* < 0.01) and loss of access primary patency (HR 2.87, 95% CI 1.57–5.27, *p* < 0.01). Stent graft procedures were performed by nine operators. In the operator-experience sensitivity analysis, additional adjustment for cumulative prior operator experience did not materially change the association between LGM and loss of stent graft patency (HR 3.16, 95% CI 2.01–4.96, *p* < 0.001).

### Exploratory subgroup analysis by access type

**Figure 3.**
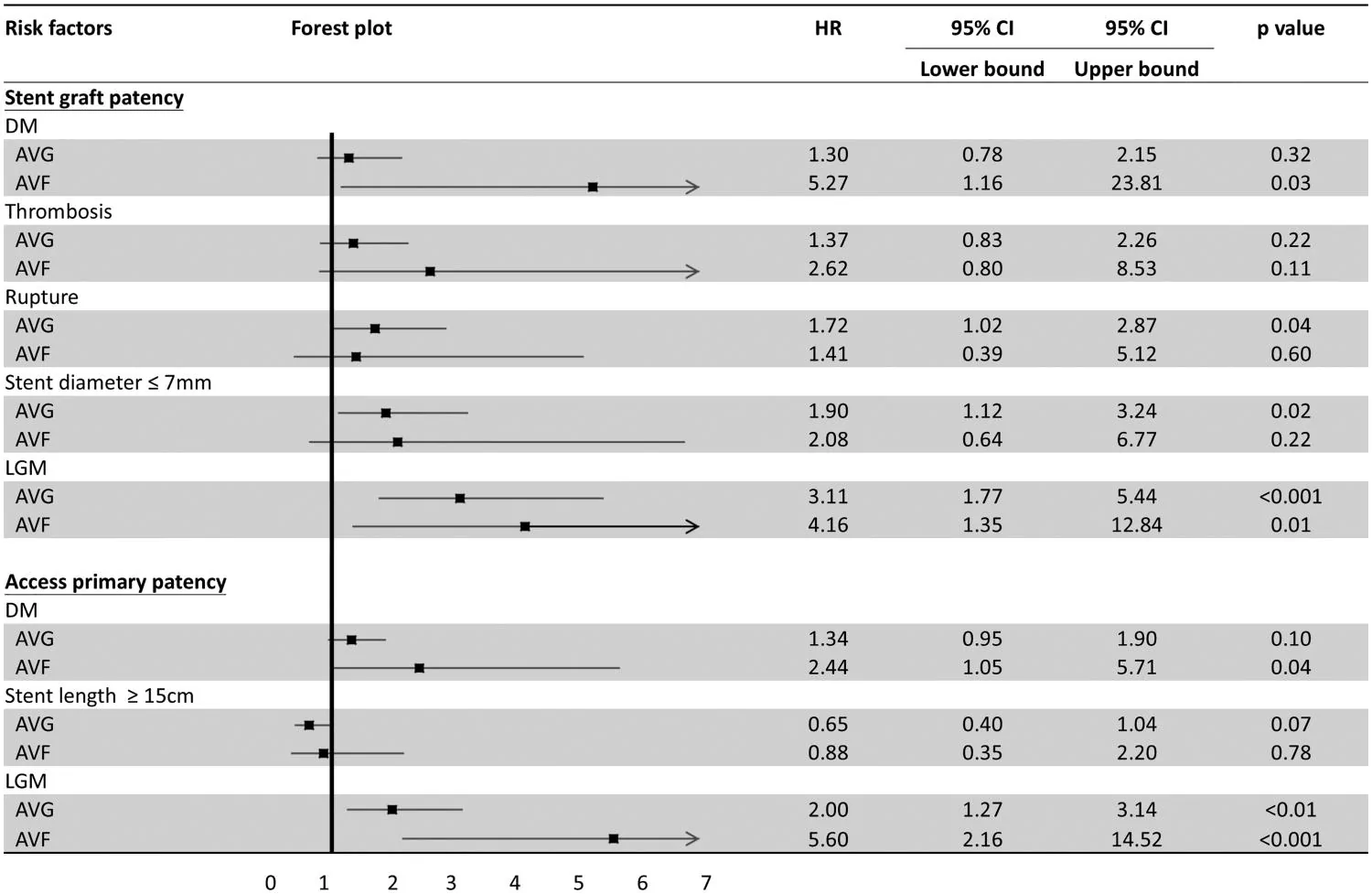
Hazard ratio of risk factors for stent graft patency and access primary patency of the arteriovenous fistula (AVF) subgroup and arteriovenous graft (AVG) subgroup. CI, confidence interval; HR, hazard ratio; LGM, longitudinal geographic miss.

Because most patients had AVG access, subgroup analyses by access type were considered exploratory (Figure 3). In the AVG subgroup, LGM was associated with loss of stent graft patency (HR 3.11, 95% CI 1.77–5.44, *p* < 0.001), as were rupture or dissection (HR 1.72, 95% CI 1.02–2.87, *p* = 0.04) and stent diameter ≤7 mm (HR 1.90, 95% CI 1.12–3.24, *p* = 0.02). For access primary patency in AVG, LGM was also associated with patency loss (HR 2.00, 95% CI 1.27–3.14, *p* < 0.01), whereas stent graft length ≥15 cm showed a nonsignificant trend toward lower risk (HR 0.65, 95% CI 0.40–1.04, *p* = 0.07).

In the AVF subgroup, point estimates for LGM were directionally similar for both stent graft patency and access primary patency. However, only 39 AVF cases were included, and confidence intervals were wide. Therefore, AVF-specific estimates should be interpreted cautiously, and no definitive conclusion can be drawn regarding differences between AVF and AVG subgroups.

## Discussion

### Main findings

In this retrospective cohort of 203 unique patients undergoing index stent graft implantation, several clinical, anatomical, and procedural factors were associated with loss of stent graft patency and access primary patency. In the primary exploratory model, loss of stent graft patency was associated with diabetes mellitus, thrombosis presentation, angioplasty-induced rupture or dissection, smaller stent diameter, and LGM. In contrast, loss of access primary patency was associated with LGM, whereas longer stent graft length was associated with a lower hazard of access primary patency loss. In the additional clinically adjusted model, LGM remained associated with both outcomes after adjustment for available lesion and procedural factors. These findings suggest that LGM is a clinically relevant technical marker and may represent a modifiable procedural target, but they should not be interpreted as evidence of direct causality.

### Technical and lesion-related factors associated with stent graft patency

Our findings suggest that technical and lesion-related factors are important correlates of stent graft patency after implantation. LGM was consistently associated with higher hazards of patency loss. Smaller stent diameter and rupture or dissection were also associated with loss of stent graft patency, possibly reflecting smaller target vessels, more complex lesions, or greater procedural injury. These observations are biologically plausible because residual edge stenosis, insufficient lesion coverage, and vessel injury may contribute to edge restenosis and repeat intervention. However, because this was an observational study, LGM should be interpreted as an associated technical marker rather than definitive evidence of a causal mechanism.

Undersizing and oversizing primarily reflect device-to-vessel diameter matching, whereas LGM reflects whether the stent graft terminates in a relatively healthy or residual diseased vessel segment. In this study, oversizing and undersizing were associated with patency loss in univariable analyses but were no longer significant after multivariable adjustment, whereas LGM remained associated with both outcomes. This pattern suggests that, for a covered stent, landing-zone vessel health may be more influential than modest diameter mismatch alone. The graft membrane may partly isolate residual disease within the covered segment, whereas disease immediately beyond the stent edge remains uncovered and exposed to access flow and vascular proliferative responses. Accordingly, the transition between the covered device and adjacent native vessel may represent a particularly vulnerable site of failure.

The LGM threshold was adapted from coronary intervention literature, in which edge-related disease was assessed within 5 mm of the stent margin and residual diameter stenosis >30% was considered relevant residual edge disease [9]. In our edge-level analysis, restenosis increased substantially when residual edge stenosis reached the 30–40% range, supporting the clinical relevance of this operational threshold in our cohort. However, this definition remains exploratory and requires external validation in hemodialysis access interventions. The >30% residual stenosis within 5 mm of the stent edge used to define LGM should not be confused with the KDOQI criterion for clinically significant dialysis-access stenosis [11]. KDOQI-based intervention generally requires >50% stenosis accompanied by clinical or hemodynamic abnormalities, whereas our threshold was used only to characterize the immediate post-deployment landing-zone condition.

These findings are consistent with prior observations in coronary interventions. In the STLLR trial, GM was associated with an increased risk of target-vessel revascularization after drug-eluting stent implantation [13]. Our results suggest that a similar concept may be relevant to hemodialysis access interventions, in which residual edge stenosis or incomplete lesion coverage may contribute to subsequent stent graft failure. In contrast, most systemic patient factors were not consistently associated with patency loss, although diabetes mellitus remained associated with stent graft failure, possibly through enhanced intimal hyperplasia [14]. Taken together, these findings support the clinical relevance of technical and lesion-related factors, while recognizing that the observational design precludes causal inference.

### Predictors of overall access patency

By contrast, access primary patency reflects circuit-level biology. Although AVG access showed a trend toward a higher hazard of patency loss, this association did not reach statistical significance in the multivariable model. LGM again emerged as a consistent risk factor, underscoring that poor device deployment compromises both device and circuit outcomes. A longer stent graft length (≥15 cm) was associated with a lower hazard of access primary patency loss, possibly because more complete longitudinal coverage allowed the device to terminate beyond the diseased segment in a healthier landing zone. However, this finding should not be interpreted as support for routine use of longer devices, because excessive coverage may compromise normal vessel or cannulation segments. These findings illustrate two different modes of failure: device restenosis at the stent edge versus circuit failure from access biology and new lesions elsewhere. Exploratory subgroup analyses suggested that the direction of association between LGM and patency loss was generally similar across AVF and AVG subgroups. However, the AVF subgroup included only 39 cases, and confidence intervals were wide. Therefore, these subgroup findings should not be interpreted as evidence of differential effects by access type. Larger studies are needed to determine whether the association between LGM and patency loss differs between fistulas and grafts.

### Patency outcomes in context

Our one-year patency outcome was broadly comparable with those reported in major stent graft trials in dialysis AVF [15] and dialysis AVG [3,4,16,17]. However, direct comparison should be cautious because most trials used protocolized follow-up, whereas our study relied on dialysis-unit monitoring and clinically indicated angiography. As a result, asymptomatic restenosis may have been under-detected, and our stent graft patency estimate should be interpreted as clinically detected, angiographically confirmed restenosis rather than protocol-detected restenosis.

### Clinical implications

Although causality cannot be inferred, our findings suggest that prevention of LGM should primarily focus on lesion delineation and landing-zone planning before stent graft deployment. Whenever anatomically feasible, the full longitudinal extent of disease should be covered, and both stent graft edges should terminate in relatively healthy vessel segments. The procedural objective should therefore be not only to cover the most severe stenosis, but also to avoid compromising device length such that the stent graft terminates within residual diseased vessel.

Once the stent graft edge has been placed within a diseased segment, additional postdilatation may improve expansion or apposition but has not been proven to eliminate the risk associated with an unfavorable landing zone. IVUS may be useful when lesion boundaries or reference-vessel dimensions are uncertain, whereas longer or overlapping stent grafts may facilitate complete coverage of selected extensive lesions. However, the routine use of IVUS, longer devices, overlap, or specific predilatation or postdilatation strategies cannot be supported by the present study, and excessive coverage may compromise normal vessel or cannulation segments. Prospective studies are needed to define the optimal imaging and deployment strategy for preventing LGM.

### Limitations

This study has several limitations. First, it was a single-center retrospective analysis, and residual confounding cannot be excluded. Although we adjusted for available lesion and procedural factors, LGM may still reflect lesion complexity, difficult landing zones, operator preference, or intentional avoidance of puncture zones rather than incomplete lesion coverage alone. Actual puncture sites, cannulation patterns, vessel tortuosity, calcification, and operator intent were not systematically recorded. Second, follow-up angiography was performed only when access dysfunction was suspected by dialysis-unit monitoring or clinical symptoms. Therefore, asymptomatic restenosis may have been under-detected, and detection bias cannot be excluded. Third, the AVF subgroup was small, with only 39 cases, and confidence intervals for AVF-specific estimates were wide. Therefore, subgroup analyses by access type were exploratory, not powered for definitive subgroup inference, and should not be interpreted as evidence of differential effects between AVF and AVG. Fourth, most procedures used Viabahn stent grafts, whereas only 17 procedures used Covera. Although stent graft brand was not associated with patency outcomes in univariable analysis and the association between LGM and both outcomes remained significant in the Viabahn-restricted sensitivity analysis, the study was not powered for brand-specific comparison. Therefore, the findings cannot be assumed to apply equally to other stent graft platforms. Fifth, heterogeneity in operator experience, technical variations, and potential learning-curve effects over the study period may have influenced the study results but could not be directly assessed. Sixth, although reproducibility testing showed high agreement for LGM classification, not all angiograms were independently reviewed by two assessors. Finally, the definition of LGM in hemodialysis access circuits has not been formally validated; therefore, our findings should be considered exploratory. However, despite the lack of formal validation, the underlying concept that residual edge stenosis may promote turbulent flow, edge restenosis, and recurrent access dysfunction provides biological plausibility for evaluating LGM in dialysis access circuits.

## Conclusion

In this retrospective cohort of patients undergoing index stent graft implantation for hemodialysis access dysfunction, LGM was strongly associated with loss of stent graft patency and access primary patency. Stent diameter, thrombosis presentation, rupture or dissection, diabetes mellitus, and stent graft length were also associated with patency outcomes. LGM may represent a potentially modifiable procedural target, but the observational design and possibility of residual confounding preclude causal inference. Prospective studies with standardized imaging follow-up and detailed assessment of lesion anatomy, puncture-zone location, and deployment strategy are needed to determine whether reducing LGM improves stent graft and access outcomes.

## Data Availability

The data that support the findings of this study are not publicly available due to institutional and patient privacy restrictions but are available from the corresponding author upon reasonable request and with permission of the Institutional Review Board.
